# The Comparison of Breast Reconstruction Using Two Types of Acellular Dermal Matrix after Breast-Conserving Surgery

**DOI:** 10.3390/jcm10153430

**Published:** 2021-07-31

**Authors:** Jeongshin An, Hyungju Kwon, Woosung Lim, Byung-In Moon, Nam Sun Paik

**Affiliations:** Department of Surgery, Ewha Womans University School of Medicine, Ewha Womans University Mokdong Hospital, 1071 Anyangcheon-ro, Yangcheon-gu, Seoul 07985, Korea; jsan@ewha.ac.kr (J.A.); hkwon@ewha.ac.kr (H.K.); limw@ewha.ac.kr (W.L.); mbit@ewha.ac.kr (B.-I.M.)

**Keywords:** breast neoplasm, breast-conserving surgery, breast reconstruction, acellular dermal matrix

## Abstract

Breast reconstruction during breast-conserving surgery (BCS) can improve the breast shape. This study introduces breast reconstruction in BCS with two types of acellular dermal matrix (ADM). The study included 134 patients who underwent BCS due to breast cancer from February 2018 to May 2021. This study was conducted by one surgeon, and is the result of a three-year study. The patient group who underwent BCS using ADM was mainly targeted at patients with minor to severe defects after the operation. The average age of the patients was 51.8 years, and the body mass index (BMI) was 23.8 kg/m. The specimen weight was 30–120 g. The average surgical time, including reconstruction, was 100.4 min, combined with reconstruction. There were minor complications in six patients. The advantage of using ADM is that it can quickly correct the shape of the breast after conventional BCS surgery. Pellet-type ADM, rather than sheet-type, can create a breast shape similar to that before surgery. Breast reconstruction using ADM can be an easy and convenient method for making a better shape from BCS.

## 1. Introduction

Breast-conservation surgery (BCS) has proven to be oncologically safe, and is currently recognized as one of the most common surgical methods in South Korea [1]. BCS has been developed to minimize defects of the breasts via oncoplastic surgery [2]. However, BCS leaves a defect when the breast is extensively resected, and the defect becomes relatively large, especially when the breast is small [3]. This study found that breast reconstruction using the acellular dermal matrix (ADM) can overcome these limitations.

In breast reconstruction, ADM is generally used as a material for covering implants [4]. It is increasingly being used in the reconstruction of nipple-sparing mastectomy and skin-sparing mastectomy via implant insertion in recent years [5,6]. How to use the ADM of breast surgery is already in place, and its safety has been established [7,8]. However, there have been rare reported cases of reconstruction using the ADM in BCS. The ADM is a useful method for the wide excision of breasts, and several methods have been devised to improve the most common problem: infection.

The authors investigated how to achieve ideal results when performing simultaneous breast reconstruction after breast-conserving surgery with two types of ADM. During surgeries, two focuses existed. The first one was the size and shape of the ADM, and the second one was to prevent infection after surgery. Several devised procedures were successful in avoiding infection. Breast reconstruction using the ADM can be an alternative to compensate for the shortcomings of BCS.

## 2. Patients and Methods

### 2.1. Patient Selection

**Figure 1:**
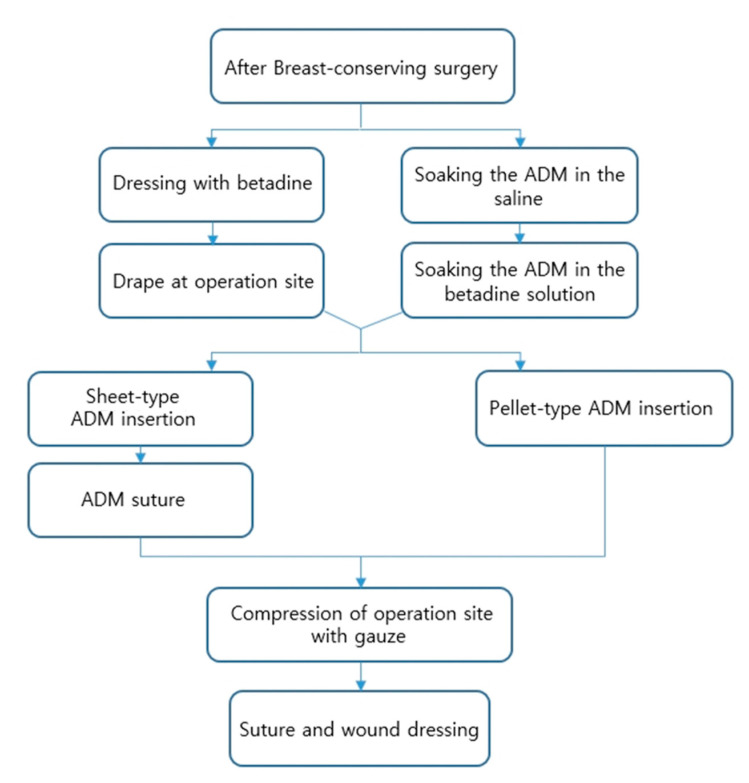
Surgical order of breast reconstruction using two types of ADM. ADM = acellular dermal matrix.

The 134 patients who underwent breast-conserving surgery were in a single institution, with a single surgeon, between February 2018–May 2021. This study was approved by the institutional review board of the Ewha Woman’s University Mokdong Hospital (IRB number: 2021-06-030). In all patients, the ADM was used as MegaDerm^®^ (L & C BIO, Seongnam, Korea) or CG CryoDerm (CGBio Corp., Seongnam, Korea), and two types were used: a sheet type or pellet type, according to surgical order (Figure 1). The data were compiled into a spreadsheet using Microsoft Excel (Microsoft Corp., Redmond, WA, USA).

### 2.2. Surgical Technique

**Figure 2:**
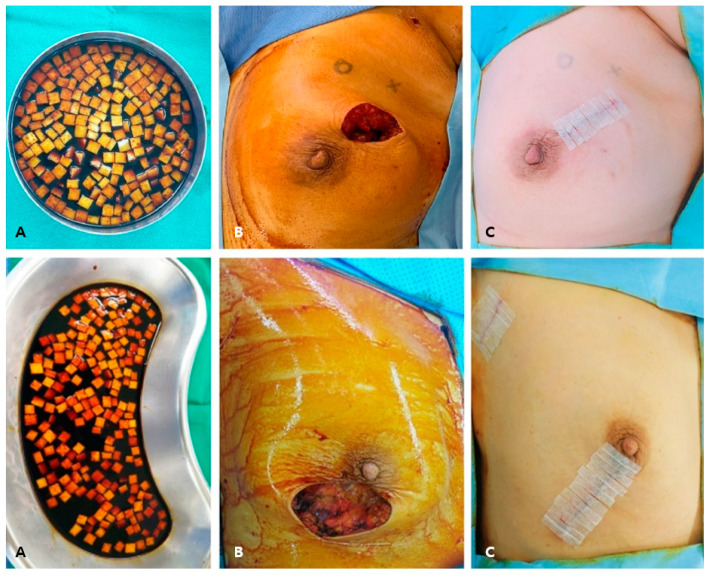
Main steps of the reconstruction using the pellet-type ADM after BCS. (**A**) The ADM was soaked in betadine solution. (**B**) Betadine dressing and re-drape was performed after breast wide excision. (**C**) The ADM was applied at the surgical cavity, and the wound was sutured. ADM, acellular dermal matrix; BCS, breast-conserving surgery.

**Figure 3:**
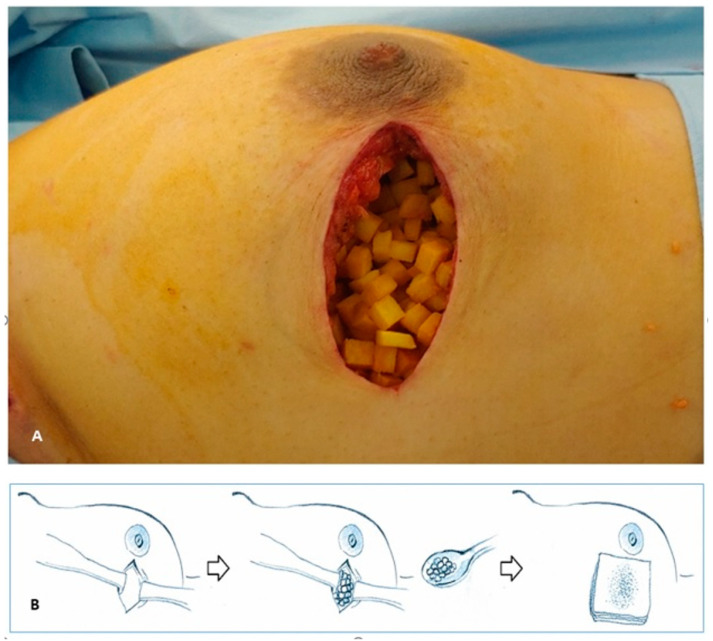
How to insert the ADM. (**A**) The ADM soaked in betadine solution was inserted at the surgical cavity. (**B**) After ADM insertion, compression with gauze was needed for removal of remnant betadine solution and discharge. ADM, acellular dermal matrix.

BCS was performed after a 3.5–5 cm skin incision. The operation to make a radial incision towards the axilla was performed with a sentinel lymph node biopsy through the same incision, and in other cases, a separate incision was made. The wide excision of the breast tumor was performed, and cavity margins were confirmed by frozen biopsy. If cavity margins were positive for malignancy, further resection was needed. If the frozen biopsy was negative for malignancy, surgical clips were applied for radiation therapy. At this time, the ADM was soaked in saline, and then in a 1:1 mixture of saline and betadine solution for 20 min (Figure 2A). The surgical site was dressed in betadine solution, and additional drapes were applied (Figure 2B). New surgical instruments were used, along with the tool used for wide resection. After that, the ADM was immersed in betadine solution, and was filled into the surgical site (Figure 2A). Since the ADM absorbed the betadine solution, the gauze was pressed on the surgical site to remove the betadine solution and perform sutures (Figure 2C). The picture of the ADM filled in the surgical site and the schematic diagram of the overall operation are as follows (Figure 3A,B). When the wide excision of breast and sentinel lymph node biopsy were performed with the same incision, the ADM could be directed toward the axilla. In that case, the axillary and chest border were separated via sutures.

## 3. Results

One hundred and thirty-four patients were included, and all patients underwent BCS. Patient characteristics are described in Table 1. The mean patient age was 51.8 years (range = 27–74 years), and BMI was 23.8 kg/m (range = 16.6–36.8 kg/m^2^). The average operation time was 100.4 min (range = 40–200 min), and the average reconstruction time was 30 min (range = 20–60 min). The sheet-type ADM was applied to 33 patients, and the pellet-type ADM was applied to 101 patients (Table 1). The drainage tube was not inserted after the operation, and aspiration was performed with a syringe after the operation if necessary. Minor complications occurred in six patients (4.5%). Minor complications were defined according to the Clavien–Dindo classification below grade III [9]. One patient had hematoma, another patient had granuloma, and three patients had surgical site inflammation (Table 2). One patient had a granuloma surgical excision; four inflamed patients were given antibiotics and the ADM was removed. The other patient recovered without special treatment. Cases using the sheet-type ADM had more complications compared to those of the pellet-type ADM.

Patients who visited the outpatient clinic after ADM reconstruction were asked about their breast shape, and all patients were satisfied with the postoperative breast shape. Occasionally, some patients complained of the feeling of a foreign body. However, all the patients agreed that the shape of the breast was similar to that before surgery. The satisfaction of the surgeon who performed the operation was also significantly better in all patients compared to the BCS without ADM insertion.

## 4. Discussion

**Figure 4:**
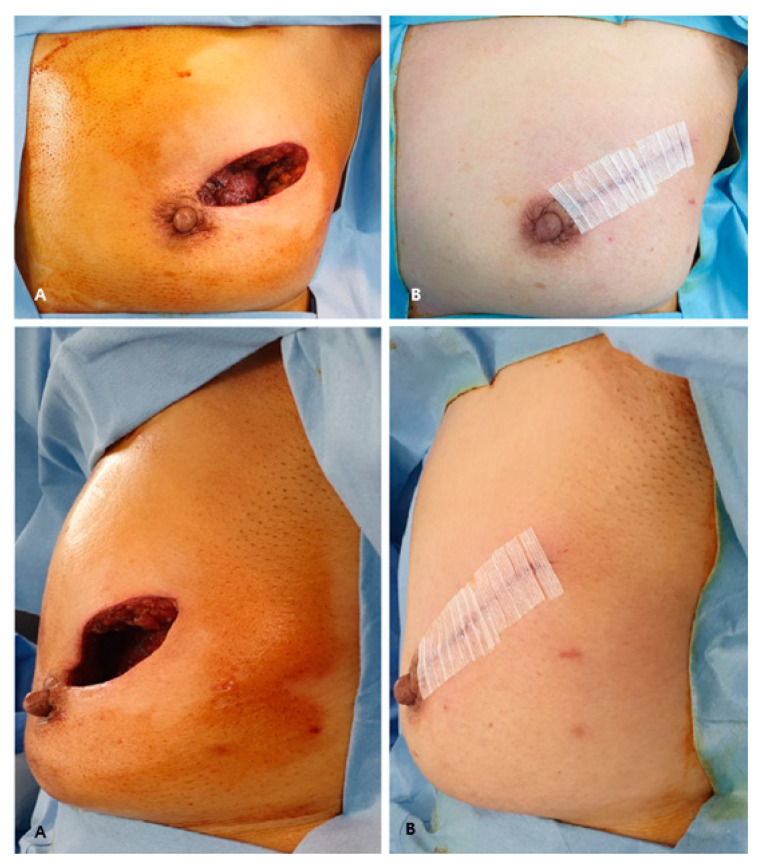
A wide excision leaves a defect; a technique that can compensate for this is reconstruction using the ADM. (**A**) These pictures show extensive surgical cavities. Top A represents the front of the cavity, and bottom A represents the side of the cavity. (**B**) ADM insertion was performed to compensate for the surgical cavity. After ADM insertion, top B shows the front of the surgical wound, and bottom B shows the side of the surgical wound.ADM, acellular dermal matrix.

**Figure 5:**
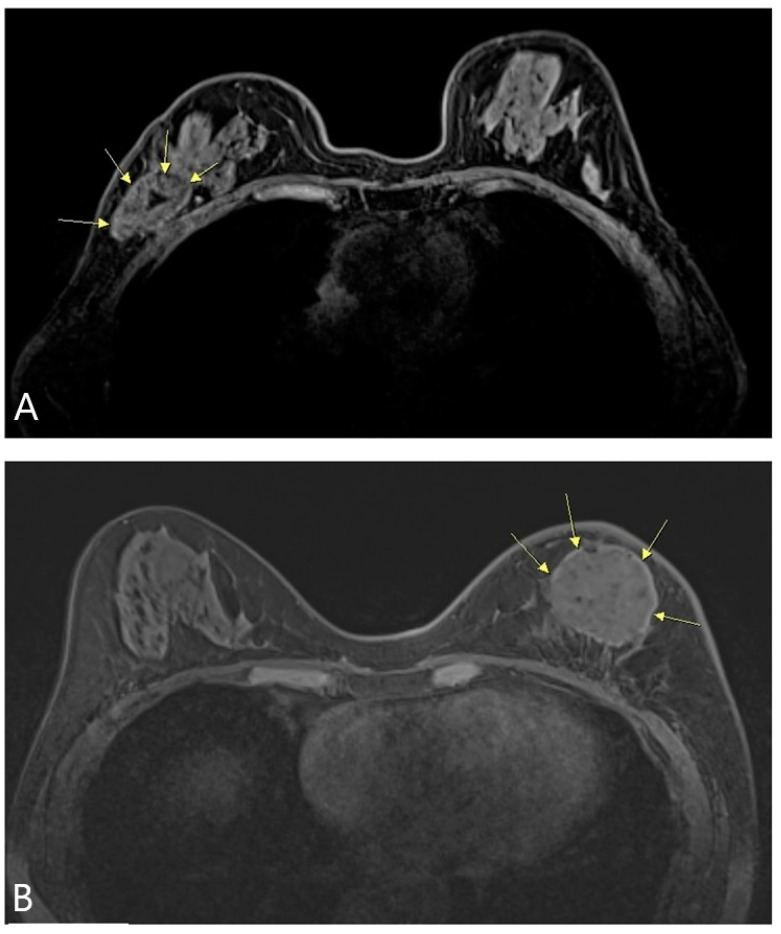
MRI on the breasts was performed after reconstruction using two types of ADM. (**A**) The sheet-type ADM (yellow arrows) was used in this BCS patient. (**B**) The pellet-type ADM (yellow arrows) was applied for reconstruction after BCS. ADM, acellular dermal matrix; BCS, breast-conserving surgery; MRI, magnetic resonance imaging.

BCS with radiation therapy has developed because of the advantage of preserving the breast shape, and shows a similar oncological outcome compared to mastectomy [10]. As seen from the first radical mastectomy and the development of modified radical mastectomy to BCS, the surgical site was gradually minimized [11]. However, in BCS, extensive resection leaves a defect (Figure 4A), and a technique that can compensate for this is reconstruction using the ADM (Figure 4B). This method can maintain the shape of the breast, keeping it similar to that before surgery. In addition, it can be reconstructed quickly (Table 1), reducing the operation time compared to other reconstruction methods. In particular, when the pellet-type ADM was used for reconstruction, the shape of the breast reconstruction was more similar to that before surgery (Figure 5A,B), and patient satisfaction was high.

There were four cases of infection, of which three cases occurred before using a secondary infection prevention method with betadine. To reduce infection, we used the betadine sterilization process, drape retry, and ADM betadine-soaking method, which significantly reduced the chance of infection. After using this method, the number of infection cases was reduced to one case. Furthermore, as a result of the interim analysis of this study, there were five cases of complication using the sheet-type ADM. This complication rate is acceptable. However, this result provided an opportunity to change all of the reconstruction cases to the pellet-type ADM. The cases of side effects were different according to ADM type. ADM types differ in the process of adjusting the shape of the surgical cavity. More side effects existed using the sheet-type ADM. The pellet-type ADM is flexible, and the sheet-type ADM can be folded to fit the shape of the surgical cavity. The sheet-type ADM is angled when folded (Figure 5A). In this process, the sheet-type ADM affects the tension on the surgical wound; therefore, the wound is easily stimulated. Excessive inflammation of the surgical site makes the wound chronic through continuous destruction of the wound tissue [12]. This process promotes tissue damage and delays wound healing. In addition, excessive inflammatory cell recruitment and biofilm formation due to bacterial infection cause a chronic wound [13]. These processes increase wound inflammation and delay wound healing, which may be associated with an increase in inflammatory cases. The pellet-type ADM is movable, making it easier to fit into surgical cavities and less inflammatory than the sheet-type ADM.

According to previous studies, diabetic mellitus (DM), smoking, and neoadjuvant chemotherapy usually cause side effects of the ADM [14]. Among the adverse events in this study, no one was diagnosed with DM, one was diagnosed with hyperlipidemia, and all of them were non-smokers. There was one case in which the ADM was removed due to inflammation during adjuvant chemotherapy. Hyperlipidemia and chemotherapy are both associated with an inflammatory response, which may be the cause of ADM side effects [15,16]. Oxidized regenerated cellulose polymer (ORCP), one of the biomaterials similar to ADM, has been used in breast-conserving surgery to make up the breast shape [17,18]. ORCP differs from the ADM in that it is absorbed into the surrounding tissues after surgery. Therefore, when ORCP is inserted, the lower the tissue density, the higher the risk of fat necrosis and the lower the effectiveness of mammoplasty [18]. On the other hand, the ADM is not absorbable material. Therefore, it is advantageous in maintaining the shape of the breast, and the ADM is a better material for breast reconstruction in this respect.

The ADM was studied to replace extensively burned skin for the first time [19]. Since then, using the ADM has evolved in various surgical fields, such as protection for wounds, tendons, bones, cartilage, and nerves [20,21], as well as the reconstruction of various organs in the human body [22]. The ADM is used for implant wrapping in nipple-sparing mastectomy and skin-sparing mastectomy nowadays, and has already secured stability. However, the method of BCS reconstruction using the ADM is rarely known [23,24]. There was no comparison according to ADM type during BCS surgery before. Therefore, this study is important, because it is the first report of BCS reconstruction depending on the ADM type.

A limitation of BCS reconstruction using the ADM is that there was one case of granuloma caused by an inflammatory response. It is not known which patients will develop the granuloma. However, since this case is rare [25], the advantage of reconstruction using the ADM is greater, and this shortage can be covered.

## 5. Conclusions

Breast reconstruction with the ADM after BCS can rapidly correct breast shape and prevent infection by following a devised surgical procedure. The advantage of the ADM is that it is easier to maintain the breast shape than other absorbent materials. Moreover, the pellet-type ADM showed better results and fewer complications after surgery, and the shape after reconstruction was similar to that before surgery. Breast reconstruction using the ADM can be an easy and convenient way to create a better shape of the breasts after BCS.

## Figures and Tables

**Table 1:** Patient characteristics.

		Total	Sheet-Type ADM	Pellet-Type ADM
Age	Mean ± SD (Range)	51.8 ± 9.3 (27–74)	52.5 ± 10.8 (31–74)	51.6 ± 8.8 (27–71)
BMI	Mean ± SD (Range)	23.8 ± 3.8 (16.6–36.8)	22.7 ± 3.8 (17.2–35.9)	24.2 ± 3.8 (16.6–36.8)
Hight	Mean ± SD (Range)	158.8 ± 5.3 (143.8–173.1)	158.5 ± 5.4 (143.8–168)	158.9 ± 5.3 (146–173.1)
Weight	Mean ± SD (Range)	60.1 ± 10.5 (42.5–97.8)	57.1 ± 10.9 (43.7–97.8)	61.1 ± 10.2 (42.5–91.7)
Stage	Range	Stage 0–IIIA	Stage 0–IIIA	Stage 0–II
Total operation time	Mean ± SD (Range)	100.4 ± 22.1 (40–200)	94.9 ± 23.6 (40–150)	101.7 ± 21.9 (55–200)

ADM, acellular dermal matrix; BMI, body mass index; SD, standard deviation.

**Table 2:** Comparison between ADM types.

	Category	Patient Number (%)
Cases	Sheet-type ADM	33 (24.63%)
	Pellet-type ADM	101 (75.37%)
Complication cases	Sheet-type ADM	5 (3.73%)
	Pellet-type ADM	1 (0.75%)
Classification of complication	Major	0
	Minor	6 (4.48%)
Types of complication	Hematoma	1 (0.75%)
	Infection	4 (2.99%)
	Granuloma	1 (0.75%)

ADM, acellular dermal matrix.

## Data Availability

Not applicable.
